# In dogs with ruptured cranial cruciate ligament, is TPLO superior to TTA in reducing postoperative radiographic osteoarthritis?

**DOI:** 10.18849/ve.v11i3.749

**Published:** 2026-07-10

**Authors:** William Grech

**Keywords:** CRANIAL CRUCIATE LIGAMENT, OSTEOARTHRITIS, MODIFIED MAQUET PROCEDURE, TIBIAL PLATEAU LEVELLING OSTETOMY, TIBIAL TUBEROSITY ADVANCEMENT

## Abstract

**PICO question:**

In dogs with a naturally occurring ruptured cranial (anterior) cruciate ligament [P] does surgical treatment using tibial plateau levelling osteotomy (TPLO) [I] compared to a surgical repair using tibial tuberosity advancement (TTA) [C] result in a reduction in the risk of developing postoperative osteoarthritis? [O]

**Clinical bottom line:**

**Category of research:**

Treatment.

**Number and type of study designs reviewed:**

Three papers were reviewed. These were a prospective, randomised, non-controlled clinical trial; a prospective, randomised, controlled clinical trial; and a retrospective, non-controlled, blinded cohort study.

**Strength of evidence:**

Weak.

**Outcomes reported:**

The first clinical trial showed that TTA was associated with less osteoarthritis (OA) progression than TPLO at 6-month follow-up. However, the opposite was reported in the second clinical trial, where less OA was recorded following TPLO than TTA surgery. In both the first and second studies, the difference between groups was not significant. The third study reports a significant increase in OA score in TTA over TPLO group at end of follow-up period. Despite the third study claiming a noticeable difference in recorded OA between these two surgeries, evidence is presently scant.

**Conclusion:**

At the time of writing of this Knowledge Summary there is minimal evidence to conclude either TTA or TPLO reduces the rate of postoperative osteoarthritis progression for dogs treated for naturally occurring cranial cruciate ligament rupture.

**Additional comments and caveats:**

All three studies possess various limitations, such as a lack of controls, absent confidence intervals, lacking blinding, small sample sizes, presence of confounders, bias, and short follow-up, all of which limit the value of their conclusions.

How to apply this evidence in practice

The application of evidence into practice should take into account multiple factors, not limited to: individual clinical expertise, patient’s circumstances and owners’ values, country, location or clinic where you work, the individual case in front of you, the availability of therapies and resources.

Knowledge Summaries are a resource to help reinforce or inform decision making. They do not override the responsibility or judgement of the practitioner to do what is best for the animal in their care.

## Clinical scenario

An eight-year-old female spayed Labrador presented with hindlimb lameness while playing. Clinical examination revealed effusion of the right stifle joint and a positive cranial drawer/tibial compression test. Radiography revealed right stifle effusion. A diagnosis of a complete cranial cruciate ligament rupture was made. The client was informed about two osteotomy techniques for the treatment of a ruptured cruciate ligament, TPLO and TTA. Both techniques are associated with the progression of osteoarthritis (Livet et al., 2019; Knebel et al., 2020; Moore et al., 2020). Is there evidence that TPLO surgery is associated with a lower progression of OA than TTA?

## The evidence

There is no clear consensus among studies reviewed about which surgery provides the best outcome in reducing development of OA (Livet et al., 2019; Knebel et al., 2020; Moore et al., 2020). Progression of OA is seen in both TPLO and TTA surgery, with two (Knebel et al., 2020 and Moore et al., 2020) out of three studies indicating that less is seen after TPLO surgery. The cohort study by Moore et al. (2020) was the only study out of three which concluded that TPLO surgery leads to a better outcome than TTA, however the difference reported in radiographic scoring and owner questionnaires, between both surgeries is not clinically meaningful after a three-year follow-up. Moreover, this study lacked a control group, and samples were not randomised. In addition, bias was present in the study with the TPLO group nearly four times the size of the TTA group. Livet et al. (2019) demonstrated that in either treatment group a rise in median OA score was observed, with TPLO dogs scoring slightly higher scores than TTA. However, these findings were not found statistically significant. In summary, there is no conclusive evidence to support the clinician that TPLO is superior to TTA in reducing radiographic progression of OA postoperatively.

**Knebel et al. (2020):** Outcome after Tibial Plateau Levelling Osteotomy and Modified Maquet Procedure in Dogs with Cranial Cruciate Ligament Rupture **Aim: **To compare outcomes in tibial plateau levelling osteotomy (TPLO)/Modified Maquet Procedure (MMP) procedures using radiographic and force plate analysis.

Study design:	Prospective, randomised, controlled clinical trial.
Interventions:	61 dogs between 20–35 kg bodyweight with cranial cruciate ligament (CCL) disease with absence of other disease were included.Treatment group: TPLO group: 30 dogs (41 stifles) and MMP group: 31 dogs (35 stifles), some had bilateral cruciate disease TPLO group mean age of dogs was 5.9 ± 2.5 years (range: 2–12.3 years). Dogs had a mean body weight of 30.0 ± 4.64 kg. MMP group mean age of dogs was 6.7 years ± 3.0 (range: 0.9–11.3 years). Participants had a mean body weight of 29.9 ± 4.8 kg. Control group (n = 16), consisted of Labrador Retrievers with a mean age of 4.1 ± 1.5 years (range: 1.7–6.7 years) and a mean body weight of 30.5 ± 5.3 kg. TPLO and MMP procedures were performed in dogs in an alternating sequence. In dogs with bilateral disease, the same procedure was performed in both stifles. Preoperative mediolateral and caudocranial radiographs were performed for all dogs in the treatment groups. Magnetic resonance imaging (MRI) performed to assess meniscal injury, with T1/T2/Proton density weighted sagittal planes acquired. Immediate postoperative caudocranial and mediolateral radiographs were performed for all dogs in either treatment group. Gait analysis consisting of Peak Vertical Force and Vertical Impulse studies performed preoperatively, 6-weeks, 3-months and > 6-months postoperatively. All patients were treated with carprofen (4.4 mg/kg) orally for 10 days. Patients were discharged home 3 days after surgery.
Outcome studied:	An orthopaedic exam: Performed initially and participants were scored for lameness (score range 0–4), presence or absence of cranial tibial thrust instability, joint swelling, and presence of pain on manipulation of stifle joint. Force plate analysis: Used to evaluate walking on a treadmill (speed: 0.9 and 1.2 m/s) for participants in both the treatment and control groups. Peak vertical force (PVI) and vertical impulse (VI) were recorded for all groups. Radiography: In both treatment groups, mediolateral and caudocranial stifle radiographs taken pre- and postoperatively as well with each follow-up at 6 weeks, 3 months, and 6 months postoperatively. Graded for OA using 1–4 scale.
Main Findings (relevant to PICO question):	Orthopaedic exam findings: MMP dogs exhibited a higher median lameness score 6 weeks postoperatively than TPLO patients, though not found to be statistically significant. At 3- and 6-month follow-up, the median lameness score for both groups improved. 4/30 of the TPLO group and 5/28 in the MMP group, experienced mild-to-moderate lameness at 6 months. Force plate analysis (PVI): For both surgical techniques studied, at each time point, neither treatment group was found to be statistically significantly different from the other, except for TPLO dogs at 3-month follow-up (P = 0.035). At this time period 21 dogs undergone TPLO achieved results that were within the ‘controls’ reference range, contrary to only 13 for the MMP group. Vertical Impulse analysis (VI) for both TPLO and MMP were not found significant at either time point. Radiography: Mild progression of OA was noticed in both TPLO and MMP group at 6 months postoperatively. However, the progression of OA in both treatment groups was not found to be significantly different from each other.
Limitations:	Small sample size in both treatment groups limit the power of the study. Radiographs of elbows, hips, tarsal joints and lumbar spine were not performed in the treatment group. Therefore, one cannot be sure if lameness persisting at the 6-month follow-up was due to stifle surgery or due to other causes. Follow-up was not long enough to assess progression of OA beyond 6 months. OA radiographic scoring did not follow a validated method. A validated owner questionnaire (e.g. Liverpool Osteoarthritis in Dogs) was not completed. Inter/intra-group variation is likely to have been introduced in this study when dogs with bilateral ruptured CCL were allowed to participate. Entry criteria for control group not defined. Control group was not radiographed at follow-up visits for ethical reasons.
Strengths:	Control group was included and dogs participating in the study had similar lameness severity to avoid bias.
Strength of evidence:	Weak.

**Livet et al. (2019):** Comparison of Outcomes Associated with Tibial Plateau Levelling Osteotomy and a Modified Technique for Tibial Tuberosity Advancement for the Treatment of Cranial Cruciate Ligament Disease in Dogs: A Randomized Clinical Study **Aim: **To compare outcomes and complications in dogs with cranial cruciate ligament (CCL) disease after tibial plateau levelling osteotomy (TPLO)/tibial tuberosity advancement (TTA) surgery.

Study design:	Randomised clinical study.
Interventions:	26 dogs over 20 kg with unilateral ruptured CCL disease were included in the study. Dogs with bilateral CCL disease and other orthopaedic conditions were excluded from the study. Participants were randomly allocated into a TPLO (n = 13) or TTA group (n = 13). The RAND function on an Excel spreadsheet was used for randomisation of samples. Prior to surgery patient signalment, gait analysis on pressure walkway and radiographs of stifle were taken for OA scoring. Surgeries were performed by either a surgery resident under supervision of a board-certified surgeon or by a board-certified surgeon. At time of intervention arthroscopy performed to assess cartilage damage. TTA group: TTA rapid cages used were as follows: 5/13 dogs 9/19 mm, 1/13 dogs 9/22 mm, 1/13 dogs 9/25 mm, 4/13 dogs 10.5/19 mm, 1/13 dogs 10.5/22 mm and finally 1/13 dogs 12/25 mm. TPLO group: Stabilisation performed with 3.5 mm standard plate (9/13 dogs), broad plate (2/13 dogs), mini plate (2/13 dogs) by Synthes TPLO plate. Anti-inflammatory medication (Firocoxib, 5 mg/kg q24h) was given orally for 2 weeks. Dogs after surgery were kept in hospital for 3 days and were scored daily for lameness and gait analysis assessed by walking on a pressure walkway. Follow-up were performed by the same board-certified surgeon at 1, 3, and 6 months. This included radiography taking, lameness scores and gait analysis.
Outcome studied:	Lameness scoring. Scoring ranged from 0 (no lameness) to 5 (continuous non-weight-bearing lameness). Non-validated questionnaire completed by owners at 6 months postoperatively. Orthopaedic exam. Surgery time. Complication rate. Gait analysis using pressure walkway. Symmetry index/total pressure recorded before surgery and on each follow-up. Radiography of operated stifle joint performed on each follow-up, to evaluate progression of OA.
Main Findings (relevant to PICO question):	Lameness scores: Gradual improvement in scores with none of dogs examined lame by 6 months. At walk and trot scores were not statistically significant at 1-, 3- and 6-month follow-up. Owner questionnaire: All owners of dogs in TPLO group were satisfied at 6 months, with no lameness reported. Only 11 out of 13 owners in TTA group were fully satisfied at 6 months. In addition, one owner reported intermittent and another one permanent lameness in their dog. Symmetry Index: Symmetric Index, a measurement between healthy and affected limb, was closer to 1 in the TTA than TPLO group a month postoperatively and in subsequent checks. No significant difference between groups was found in each metric used in gait analysis. Radiographic findings: In the TPLO group an 87.5% median increase in OA was found and a 50% median increase in OA was noticed in the TTA group recorded at 6-month follow-up. The study likely lacked the statistical power to declare the observed numerical difference as significant. In both groups the increase was statistically significant from the start of study. No difference has been reported in the progression of OA between TPLO and TTA groups.
Limitations:	Small sample sizes. Short follow-ups – up to 6 months reported. Radiographs of contralateral limb not performed to compare with treated limb. OA radiographic scoring did not follow a validated method. No details were reported for the orthopaedic exam**.** No validated questionnaires used to assess owner feedback (e.g. Liverpool Osteoarthritis in Dogs) No control group.
Strengths:	Dogs participating in study had similar demographics and severity in OA/lameness to avoid bias and group variability.
Strength of evidence:	Weak.

**Moore et al. (2020):** Extended long-term radiographic and functional comparison of tibial plateau leveling osteotomy vs tibial tuberosity advancement for cranial cruciate ligament rupture in the dog **Aim: **To objectively assess osteoarthritis (OA) progression in dogs undergoing tibial plateau levelling osteotomy (TPLO)/ tibial tuberosity advancement (TTA) surgery over a 3-year period.

Study design:	Retrospective clinical cohort study.
Interventions:	TPLO group consisted of 94 dogs (39 of which had surgery in both stifles). TTA group consisted of 24 dogs (9 of which had surgery in both stifles). Past medical records from June 2012 to May 2015 were retrieved and cases of dogs with cranial cruciate ligament rupture that have undergone either TPLO or TTA were collected. Dogs with under 15 kg bodyweight or dogs that undergone arthrotomy or arthroscopy at surgery were excluded from study. Moreover, patients that had history of patellar luxation, TPLO with an inappropriate rotation or TTA with improper advancement were excluded. Participants in this study were assigned to either one of the procedures with decision based on surgeon and client preference rather than randomly determined. Radiographs performed at 8 weeks and ≥ 3 years postoperatively (mediolateral 90/90°, mediolateral 135° extension and craniocaudal projections) were gathered. All radiographs were scored using cranial ‘Cruciate ligament rupture OA scoring system’ (scale 0–5) by a board-certified radiologist. The reviewer was blinded to patient identification, history and clinical improvement for each dog. All owners completed ‘Canine Brief Pain Inventory’ (CBPI) and ‘Canine Orthopaedic Index’ (COI) questionnaires at final check-up.
Outcome studied:	Progression of OA assessed by reviewing preoperative, 8 weeks and ≥ 3 years postoperative radiographs (mediolateral and craniocaudal projections). Patient comfort and gait assessment was assessed using CBPI and COI questionnaires.
Main Findings (relevant to PICO question):	Progression in OA occurred with each follow-up whether they had undergone TPLO or TTA surgery. OA score increased more in dogs that had undergone TTA surgery or bilateral stifle surgery than in dogs that underwent TPLO surgery or were operated on only one limb. OA progression in TPLO and TTA groups was significantly different (P < 0.05) by the end of follow-up in both dogs that undergone unilateral and bilateral cruciate repair. TPLO was superior to TTA in reducing progression of OA. Owner assessment at final check-up showed that dogs that underwent TPLO surgery experienced less pain and had better function than dogs that went TTA surgery (P < 0.003).
Limitations:	No control group consisting of dogs without the disease was included. Retrospective nature of study resulted in small TTA group size studied. Being a retrospective study resulted in dogs being lost to follow-up or being deceased. The study could not be randomised. Variables like duration of clinical signs, medication history, patient history, and diet, could not be controlled, thereby increasing variation in the study. Diagnostic imaging was not performed in these dogs to confirm absence of other orthopaedic conditions, for instance hip dysplasia, that might have affected lameness in postoperative checks. OA radiographic scoring did not follow a validated method. Arthroscopic examination of stifles was not done at the time of intervention to assess for cartilage damage that might have not been identified on radiography. Advanced imaging (e.g. Computed Tomography or Magnetic Resonance Imaging) might have allowed a more thorough assessment of osteoarthritis, especially in the last follow-up. Force plate was not used to assess patient gait, rather, validated owner questionnaires were used. Although these quantitative measures of pain are used to evaluate dogs with OA, they do not give an indication of how much weight bearing is occurring in each pelvic limb.
Strengths:	Long follow-up period, with participants having similar demographics and OA scores preoperatively to avoid bias.
Strength of evidence:	Weak.

### Appraisal, application and reflection

The literature search retrieved three studies (Livet et al., 2019; Knebel et al., 2020; Moore et al., 2020) that were found pertinent in examining whether tibial plateau levelling osteotomy (TPLO) is better than tibial tuberosity advancement (TTA) surgery in reducing progression of osteoarthritis (OA). Appropriate methods employed in the above studies to address this clinical question were radiography (Livet et al., 2019; Knebel et al., 2020; Moore et al., 2020), kinetic gait analysis (Livet et al., 2019; Knebel et al., 2020) and client questionnaires (Livet et al., 2019; Moore et al., 2020). In Knebel et al. (2020), a comparison is made between TPLO and Modified Maquet Procedure (MMP), a modification of the TTA procedure. Livet et al. (2019) and Knebel et al. (2020) followed participants for only 6 months, while in Moore et al. (2020) the follow-up was more than 3 years.

All studies reported the progression of OA on radiography for both TPLO and TTA groups. In Livet et al. (2019), OA was seen to advance more in the TPLO than the TTA group with no significant difference found between categories. On the contrary, in the Knebel et al. (2020) study, MMP (TTA) group showed more progression in OA than TPLO group. However, in this study only medians and ranges were provided, and no p-values were reported on the OA follow-up results. Notably, Moore et al. (2020) showed a significant difference in OA scoring between the TPLO and TTA group, with TTA associated with more progression of OA than its counterpart. Despite the discovery of significance, the difference in means between groups is infinitesimal, thus concealing any significance there might be between the two surgeries. Despite all studies report progression in OA, Knebel et al. (2020) and Livet et al. (2019) observed no difference in limb function between the two operative methods using objective methods (e.g.: force plate analysis) at end of follow-up. Moore et al. (2020) claim that clients observed better limb function in dogs undergoing TPLO than TTA, however this is based on a subjective evaluation (questionnaire). Therefore, overall, there is poor correlation between documented radiographic OA progression and observed clinical outcome between the two surgeries.

In Knebel et al. (2020) and Livet et al. (2019) postoperative complications were reported that required partial meniscectomy. Some argue that partial meniscectomy is known to lead to the development of OA (Cox et al. 1975; Pozzi et al., 2008). Others claim the contrary (Rayward et al., 2004; Ertelt & Fehr, 2009). Moreover, in the Moore et al. (2020) study, the meniscus was released in 66/133 (49.6%) stifles at the time of surgery. A prospective, blinded, controlled, in-vivo experimental study has shown that meniscal release in dogs resulted in radiographic evidence of OA (Luther et al., 2009). Some of the reported OA progression might have occurred due to meniscal release performed intraoperatively and irrespectively of surgical method. Therefore, such procedures can have a confounding effect on the progression of OA and in any future studies it is best that dogs with meniscal lesions are excluded.

A control group was only included in the Knebel et al. (2020). A lack of controlled clinical trials, apart from Knebel et al. (2020), weakens the purported conclusions as progression in OA cannot unequivocally be attributed solely to the risk factor (the surgical technique).

Dogs over 15 kg in bodyweight took part in all three studies and in each treatment group dogs were matched for age, weight, and gender to ensure treatment groups are not dissimilar from each other. It is worth noting that in Knebel et al. (2020), dogs over 35 kg were absent. It therefore limits the application of its conclusions in giant breeds, as progression in OA might not be the same between two methods as observed in studied population due to larger forces acting on the articular surface.

Furthermore, in the Moore et al. (2020) cohort study, the TPLO group was nearly four times larger than the TTA group. This contributed to bias towards the TPLO group.. Moreover, the selection of procedure for each participant was determined by surgeon or owner preference, once again another source of bias. The non-random allocation of patients for either of the two treatment groups resulted in uneven representation of patient variables. This might have introduced bias as for instance surgeon’s bias for the TPLO surgical technique might have influenced the results.

Although the Knebel et al. (2020) clinical trial was randomised, patient selection for either procedure was performed ’alternately, in sequence’ rather than using computer software as performed by Livet et al. (2019). The former does not allow equal probability of allocation of treatment to an individual independently of previous assignment. Therefore, allocation bias might have been introduced in dog selection for next treatment. Another point of contention in Knebel et al. (2020) is that dogs with bilateral cruciate disease were compared by selecting the limb with the least signs of OA, thus omitting the effects observed in the contralateral limb. Consequently, dogs showing more progression in OA in both categories have been omitted thus the reported OA progression might have been underestimated. Dogs with bilateral disease should have been excluded from the study.

In Moore et al. (2020), the reviewer was blinded of clinical outcome when assessing follow-up radiographs, whereas in Livet et al. (2019) and Knebel et al. (2020) it was not stated if the investigators were similarly blinded of follow-up results.

A weakness in Livet et al. (2019) is that the owner questionnaire was not validated for assessing OA in dogs. In the Knebel et al. (2020), a validated owner questionnaire was not included in the study which makes collation of evidence with Moore et al. (2020) and Livet et al. (2019) difficult. On the contrary, in Moore et al. (2020) a validated clinical metrology questionnaire demonstrated the outcome in TPLO candidates to be significantly better in patient comfort than its comparison. Yet, the retrospective nature of study increases the risk of recall bias, thus participants might have left out details when reporting.

A deficiency seen in all studies is that no confidence intervals were reported for OA scores, rather means/medians and standard deviation/ranges were tabulated. Had confidence intervals been reported this would have given us more certainty about the true population value, thus increasing study validity. Moreover, in none of the studies were sample size calculations performed to determine appropriate participant numbers required to reach the desired power of the study. A difficulty noticed in comparing the two surgical methods is the inter-surgical variability inherently present with TTA. The TTA technique used in the Knebel et al. (2020) study was MMP, in which the tibial advancement is achieved using a titanium wedge while in Livet et al. (2019) a TTA cage is used. These differences increase variability within the TTA category thus making comparisons difficult.

A limiting factor compounding further ability to compare the evidence between the three studies is that the populations are different. The population in Moore et al. (2020) was young/adult with more senior dogs included in other studies, thus adding another source of variation between studies.

Despite only one study (Moore et al. 2020) claiming a noticeable difference in recorded OA between the two surgeries, presently evidence is scant, with most studies (Knebel et al. 2020 and Moore et al. 2020) reporting less OA advancement after TPLO surgery.

Finally, the studies reviewed have various limitations, namely a lack of control groups in some, a lack of blinding, small sample sizes, absent confidence intervals, presence of confounders, study bias, and short follow-up, all reducing ability to translate this information to clinical practice. In practice recommending TPLO over TTA must be based on other factors (e.g. surgeon’s preference) other than the risk of postoperative OA.  A multi-centre, randomised, controlled, clinical trial with a five-year follow-up comparing both procedures is required to investigate if TPLO results in less OA progression than TTA. Long-term follow-up will enable investigators to determine if the radiographic changes reported here similarly continue to progress in both categories and if a significant difference develops between the observed changes of both categories of procedure over time.

The conclusion of this Knowledge Summary is that there is weak evidence to support superiority of TPLO over TTA in reducing the progression of OA in dogs with cruciate rupture.

## Methodology

**Search Strategy:** 

Databases searched and dates covered:	CAB Abstracts accessed via CABI Digital Library website 2006–2025PubMed accessed via the NCBI website 2007–2025
Search strategy:	CAB Abstracts:(Canine* OR dog OR bitch*) AND (cruciate ligament OR CrCL) AND (cranial OR anterior) AND (TTA OR MMP OR Tibia tuberosity advancement) AND (TPLO OR Tibial plateau levelling osteotomy OR osteotomy) OR (“TPLO vs TTA”) AND (Osteoarthritis OR OA OR Arthritis OR DJD OR "Progression of OA" OR "functional outcome" OR radiology OR radiography OR radiographic OR "lameness score" OR "gait analysis" OR "force plate" OR kinetic)PubMed:(Canine* OR dog OR bitch*) AND (cruciate ligament OR CrCL) AND (cranial OR anterior) AND (TTA OR MMP OR Tibia tuberosity advancement) AND (TPLO OR Tibial plateau levelling osteotomy OR osteotomy) OR (“TPLO vs TTA”) AND (Osteoarthritis OR OA OR Arthritis OR DJD OR "Progression of OA" OR "functional outcome" OR radiology OR radiography OR radiographic OR "lameness score" OR "gait analysis" OR "force plate" OR kinetic)
Dates searches performed:	18 October 2025

**Exclusion / Inclusion Criteria:** 

Exclusion:	Experimental studies Duplicates Case reports Non-comparative studies Language other than English In vitro studies Biomechanical studies Systematic reviews or survival studies
Inclusion:	Peer reviewed publications Primary sources of information Canine species Studies including radiographic and lameness or kinetic studies Naturally occurring cranial cruciate disease

**Search Outcome:** 

Database	Number of results	Excluded – not primary source	Excluded – not in English	Excluded – non-comparative	Excluded – systematic review or literature review or survival analysis	Excluded – radiographic study not included to follow OA progression	Excluded – induced cranial cruciate disease	Excluded – biomechanical studies	Excluded – other species than dog	Total relevant papers
CAB Abstracts	54	5	12	10	6	10	0	8	1	2
PubMed	58	0	1	33	5	3	2	10	1	3
Total relevant papers when duplicates removed										3

## ORCiD

William Grech: 0009 0009 6986 1670

## Conflict of Interest

The authors declare no conflicts of interest.

## Disclaimer

Knowledge Summaries are a peer-reviewed article type which aims to answer a clinical question based on the best available current evidence. It does not override the responsibility of the practitioner. Informed decisions should be made by considering such factors as individual clinical expertise and judgement along with patient’s circumstances and owners’ values. Knowledge Summaries are a resource to help inform and any opinions expressed within the Knowledge Summaries are the author's own and do not necessarily reflect the view of the RCVS Knowledge. Authors are responsible for the accuracy of the content. While the Editor and Publisher believe that all content herein are in accord with current recommendations and practice at the time of publication, they accept no legal responsibility for any errors or omissions, and make no warranty, express or implied, with respect to material contained within. For further information please refer to our Terms of Use.
